# Big toenail and hair samples as biomarkers for fluoride exposure – a pilot study

**DOI:** 10.1186/s12903-019-0776-7

**Published:** 2019-05-13

**Authors:** Selma Elekdag-Turk, Mohammed Almuzian, Tamer Turk, Marilia Afonso Rabelo Buzalaf, Ali Alnuaimi, Oyku Dalci, M. Ali Darendeliler

**Affiliations:** 1 Department of Orthodontics, Faculty of Dentistry, Ondokuz Mayis University, 55139 Atakum, Samsun, Turkey; 2 Discipline of Orthodontics, Faculty of Dentistry, University of Sydney, Sydney, Australia; 3 Department of Biological Sciences, Orthodontics and Public Health, Bauru Dental School, University of São Paulo, Bauru, Brazil; 4 Department of Dentistry and Oral Health, La Trobe Rural Health School, Victoria, Australia

**Keywords:** Big toenail, Hair, Fluoride exposure, Biomarker

## Abstract

**Background:**

Biomarkers can aid in detecting and preventing clinical disease through the recognition of change in biological samples. The objective of this case-control study was to further the knowledge on the use of big toenail and hair samples as biomarkers for fluoride exposure.

**Methods:**

A total of 48 participants from an endemic (IC) and a non-endemic (SC) fluorosis region were included. Big toenail and hair samples were collected from each participant and the fluoride concentration was determined. The results of 42 participants were reported: 21 participants (11 males and 10 females, mean age 15.66 + 2.61 years) from IC and 21 participants (11 males and 10 females, mean age 15.06 + 0.79 years) from SC.

**Results:**

The mean fluoride concentration of big toenail (2.34 ± 0.26 mg/kg) and hair (0.24 ± 0.04 mg/kg) in the endemic region was significantly higher than the mean fluoride concentration of big toenail (0.98 ± 0.08 mg/kg) and hair (0.14 ± 0.02 mg/kg) in the non-endemic region (*p* < 0.001 and *p* = 0.004, respectively). The Receiver Operating Characteristic (ROC) analysis showed that the Area Under the Curve (AUC) value was 0.889 for big toenail (p < 0.001) and 0.762 for hair (p = 0.004) samples. The fluoride assay for big toenails exhibits greater observed accuracy than does the fluoride assay for hair.

**Conclusion:**

Nail and hair samples can serve as biomarkers to detect biological fluoride exposure according to the data of this pilot study. Nevertheless, hair is less sensitive and specific as a biomarker when AUC values of big toenail and hair samples were compared.

## Background

Biomarkers, i.e. biological markers, can aid in detecting and preventing clinical disease through the recognition of change in biological systems or samples [1]. Specifically, they have been defined as “cellular, biochemical, or molecular alterations which are measurable in biological media such as human tissues, cells, or fluids and are indicative of exposure to environmental chemicals” [1], such as fluoride.

Bone [2, 3], dentin [3, 4], plasma, saliva and urine [5–7] as well as nails [3, 8–12] and hair [3, 13, 14] are biomarkers that were investigated to assess fluoride exposure. It has been pointed out that among the mineralized tissues, bone is the main site for fluoride accumulation, thereby making bone a good choice as a fluoride biomarker [3]. However, the difficulty and the invasiveness of bone sample collection has been underlined. Thus, the collection of dentin, from extracted third molars, has been suggested as more appropriate when compared to bone sample collection [3].

Less invasive methods, using body fluid samples, such as plasma, saliva and urine also have been considered in the literature to analyze body fluoride concentration [5–7]. Nevertheless, these body fluids are affected by a number of variables, such as fluoride intake within the last few hours. Consequently, these body fluids present short-term, i.e. ‘snapshot’, information only [3, 7].

Nail samples, on the other hand, can be obtained non-invasively. They can be easily transported and stored for long periods of time without degradation [3, 8–11]. Nails, particularly from the big toe (hallux), providing enough mass for fluoride analysis as well as their faster growth rate when compared to the other toenails, have been recommended as suitable biomarkers for fluoride intake [3, 10]. Also, toenails have been reported to be less prone to external contaminants when compared to fingernails [3, 10, 11].

The rationale for the use of hair as a suitable biomarker for fluoride is the same as that for toenail and fingernail clippings [3]. The endogenous trace element composition of hair and nails is believed to reflect the metabolic milieu during their formation [15]. The concentration of fluoride in hair as well as nail samples represents the average level of fluoride intake and plasma concentration over an extended period of time [3, 8, 9, 13].

It was pointed out that some individuals may not accept hair sample collection, particularly individuals with long hair, since hair sample collection has to be carried out as close as possible to the scalp [3]. In these individuals big toenail clipping samples might serve as an alternative.

Different methodologies have been used for the determination of fluoride in biological materials, such as nail and hair samples. Yet, the most popular analytical method for the extraction of fluoride from biological samples has been reported to be the hexamethyldisiloxane (HMDS)-facilitated diffusion method. This technique has been described as simple and fast [8, 9, 16].

The aim of this case-control study with a 1:1 allocation ratio was to test the null hypothesis that big toenail and hair samples of subjects, living in endemic and non-endemic fluorosis regions, cannot serve as biomarkers for fluoride exposure.

## Methods

### Study sample

Sample size calculation was performed using Pocock’s formula for two means [17]. With 20 participants per group, the trial has 80% power to detect a clinically meaningful difference of 1.25 mg per kilogram (mg/kg) of fluoride in big toenail (hallux) clippings between the two regions at the 5% significance level. In order to overcome drop out and exclusion from the study, 24 participants were included in each arm of the study.

Ethical approval was granted by the Medical Faculty Ethics Committee of Ondokuz Mayis University, Turkey (No. 2008/143). The study sample included forty-eight participants who came from two regions, i.e. an endemic and non-endemic fluorosis region. The trial was undertaken with the understanding and written consent of each participant/guardian. All participants stated that they were lifelong residents in their respective areas. No changes in the address of residency (< 3 months) particularly before the commencement of this study and no history of systemic or topical fluoride supplements existed. Twenty-four participants (12 males and 12 females, mean age 15.42 + 2.50 years) were from Isparta city (IC), in the southwestern part of Turkey, with a high fluoride concentration in the public water supply (≥2 ppm (ppm)) [18, 19]. The participants from IC had Thylstrup and Fejerskov Fluorosis Indices (TFI) ranging from 2 to 5 [20]. The remaining 24 participants (12 males and 12 females, mean age 15.15 + 0.96 years) came from Samsun city (SC), on the north coast of Turkey, which has a low fluoride concentration (≤0.05 ppm) in the public water supply [19]. The Thylstrup and Fejerskov Fluorosis Indices (TFI) were 0 for the participants form SC [20]. Criteria for selection of big toenail clipping and hair samples are given in Table 1.

**Table 1:** Criteria for selection of big toenail and hair samples

Criteria for selection of big toenail samples	
• No dermatological disease, trauma or injury affecting the nails. • No nail polish or any other chemicals on nails for at least three months before the date of nail sample collection. • The patients were notified about the study in advance and advised not to cut their nails for four weeks before sample collection. Patients who had nail polish or any other chemicals on their nails waited for three months and then one extra month for nails to grow before sample collection, i.e. four months.	
Criteria for selection of hair samples	
• No dermatological disease affecting the hair. • No dyed or bleached hair. Hair was free of creams, oil and gels before sample collection. • The patients were informed six weeks in advance about the study and instructed about the dates for hair sample collection and also advised that they should not get a hair-cut during this time. If a patient has had a hair-cut, perm or colouring during the last six weeks, sample collection was postponed for six weeks as hair grows at about 0.4 mm/day or an average of about 1 cm/month.	

### Sampling and analyses

At the beginning of this study, the participants were instructed to let their big toenails and hair grow prior to sample collection for four weeks and six weeks, respectively.

On the scheduled day, the participants/parents visited their respective dental school to enable the investigator (SET) to collect big toenail and hair samples. Occipital hair is the only source recommended for the analysis for both male/female subjects. High-grade stainless steel scissors were utilized to cut the hair. The hair tuft sample was collected at a distance of 0.5 centimeters (cm) from the scalp and approximately 3 cm in length. The weight required for the hair specimen was 50–100 milligrams (mg). Hair samples were stored in labelled polyethylene bags, in a dry place, at room temperature. Again, the investigator (SET) clipped the big toenail’s free end and cut from the right side to the left side using each participant’s nail clipper. The clipped nails were stored separately in labelled plastic boxes per participant. The nail and hair samples were initially forwarded to the University of Sydney, Australia. Subsequently, the samples were sent to Bauru School of Dentistry, University of São Paulo, Brazil for analyses.

Fluoride concentration in nail and hair samples was determined after overnight HMDS-facilitated diffusion, applying the Taves method [21] as modified by Whitford [16] using a fluoride ion-specific electrode (Orion Research, Cambridge, Mass., USA, model 9409) and a miniature calomel reference electrode (Accumet, No. 13–620–79), both coupled to a potentiometer (Orion Research, model EA 940). All readings were made in duplicate.

### Statistical analysis

Statistical analyses were performed with SPSS 23.0 for windows. The mean repeatability of the readings, based on duplicate samples, was 96%. Data were presented as mean ± standard error (SE) and median (Semi Interquartile Range – SIQR). The Shapiro–Wilk test was used to analyze the normal distribution assumption of the quantitative outcomes. Mann-Whitney test was used to compare the fluoride concentration in big toenail as well as hair samples between endemic and non-endemic regions. The receiver operating characteristic (ROC) curve was used to illustrate and evaluate the performance of big toenail and hair samples as biomarkers in case of fluoride exposure. The area under the ROC curve (AUC) was evaluated as the measure of a diagnostic test’s discriminatory power. Confidence intervals can be computed for AUC. In this article, sensitivity and specificity values were evaluated. A *p* value less than 0.05 was considered as statistically significant.

## Results

The fluoride concentration in 6 participants could not be detected (3 from each city) due to technical difficulties, i.e. the fluoride concentration was not within the detection limit of the electrode since the amount of the sample collected was insufficient. Thus, 21 residents from IC (11 males and 10 females, mean age 15.66 + 2.61 years) and 21 residents from SC (11 males and 10 females, mean age 15.06 + 0.79 years) remained for the final analyses.

**Fig. 1:**
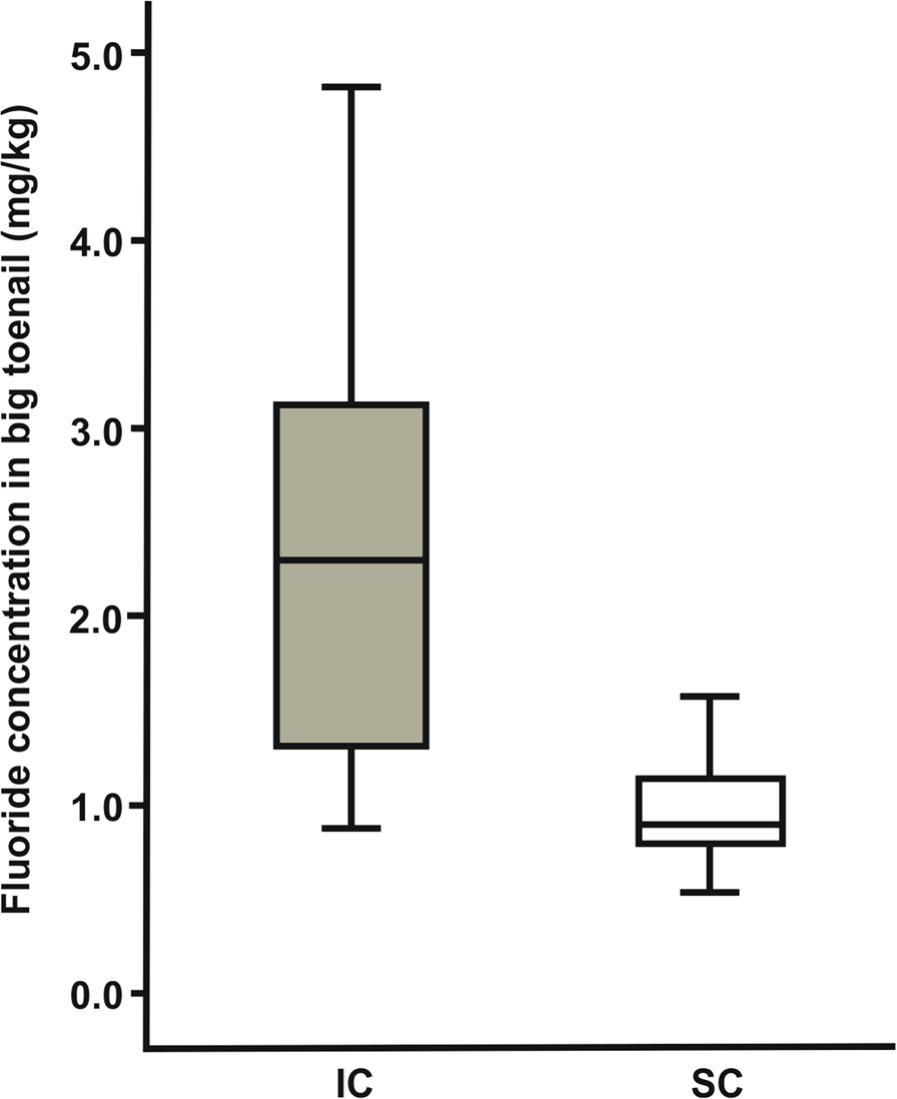
Box plots showing fluoride concentration in big toenail samples

**Fig. 2:**
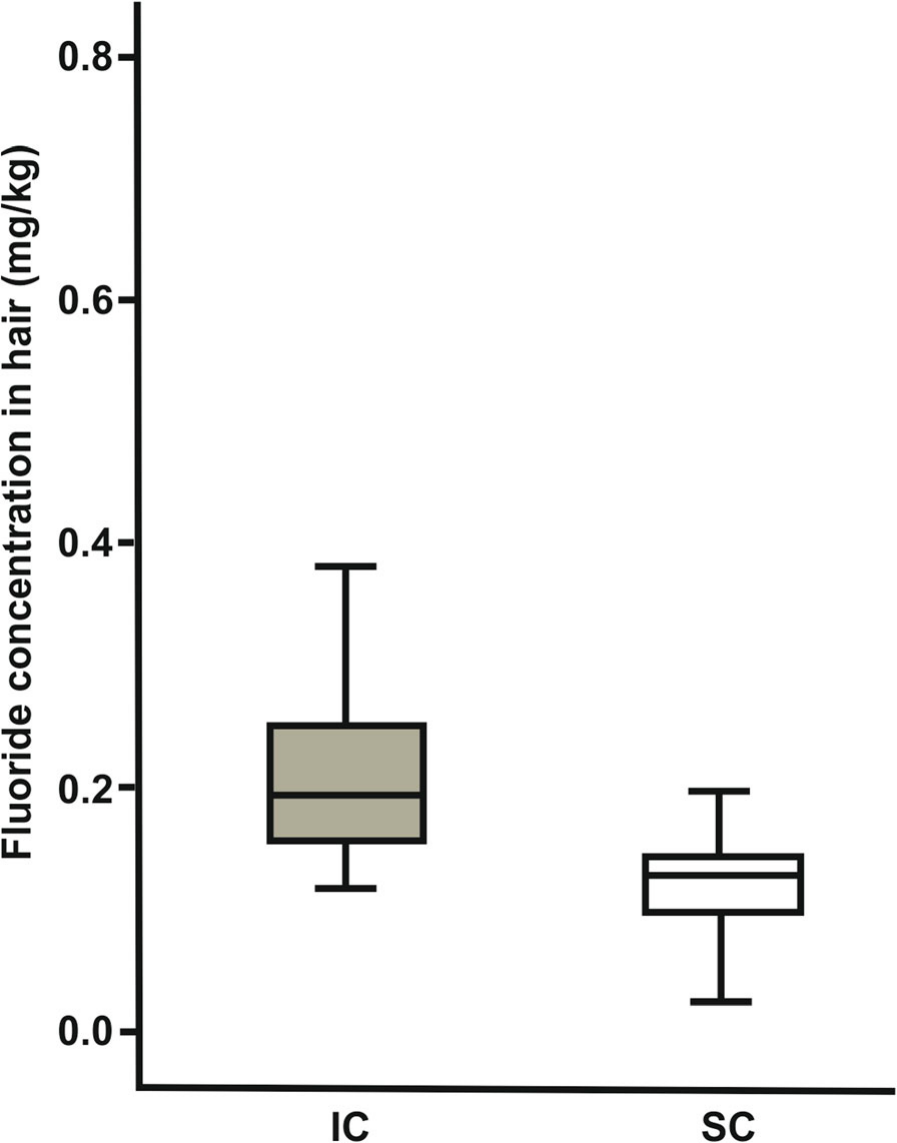
Box plots showing fluoride concentration in hair samples

Overall, the mean fluoride concentration collected from nail clippings of the residents of IC was 2.34 + 0.26 mg/kg, while it was 0.98 + 0.08 mg/kg for those from SC (Table 2). Hair sample analysis of the participants from IC displayed a fluoride concentration of 0.24 + 0.04 mg/kg, while it was 0.14 + 0.04 mg/kg for those from SC (Table 2). There was a significant difference in the concentration of fluoride in nail clipping (*p* < 0.001; Table 3; Fig. 1) and hair (*p* = 0.004; Table 3; Fig. 2) samples between the two cities, higher in participants from IC than those from SC.

**Table 2:** Fluoride concentration in big toenail and hair samples

Biomarker		IC	SC	Difference
Nail Fluoride concentration (*N* = 21) (mg/Kg)	Mean ± SE	2.34 + 0.26 (95% CI 1.80–2.88)	0.98 + 0.08 (95% CI 0.82–1.14)	1.36 + 0.25 (95% CI 0.85–1.88)
	Median	2.296 (SIQR 0.917)	0.914 (SIQR 0.189)	1.426 (SIQR 0.933)
Hair Fluoride concentration (*N* = 21) (mg/Kg)	Mean ± SE	0.24 + 0.04 (95% CI 0.17–0.32)	0.14 + 0.02 (95% CI 0.10–0.17)	0.11 + 0.04 (95% CI 0.03–0.19)
	Median	0.194 (SIQR 0.059)	0.126 (SIQR 0.045)	0.073 (SIQR 0.085)

*SE* Standard Error, *CI* Confidence Interval for Mean, *SIQR* Semi Interquartile Range for Median

**Table 3:** Comparison of fluoride concentration in big toenail and hair samples between IC and SC

Biomarker	City	N	Mean Rank	Sum of Ranks	U	P
Nail Fluoride concentration (mg/Kg)	IC	21	29.67	623.00	49.00	< 0.001
	SC	21	13.33	280.00		
Hair Fluoride concentration (mg/Kg)	IC	21	27.00	567.00	105.00	=0.004
	SC	21	16.00	336.00

**Fig. 3:**
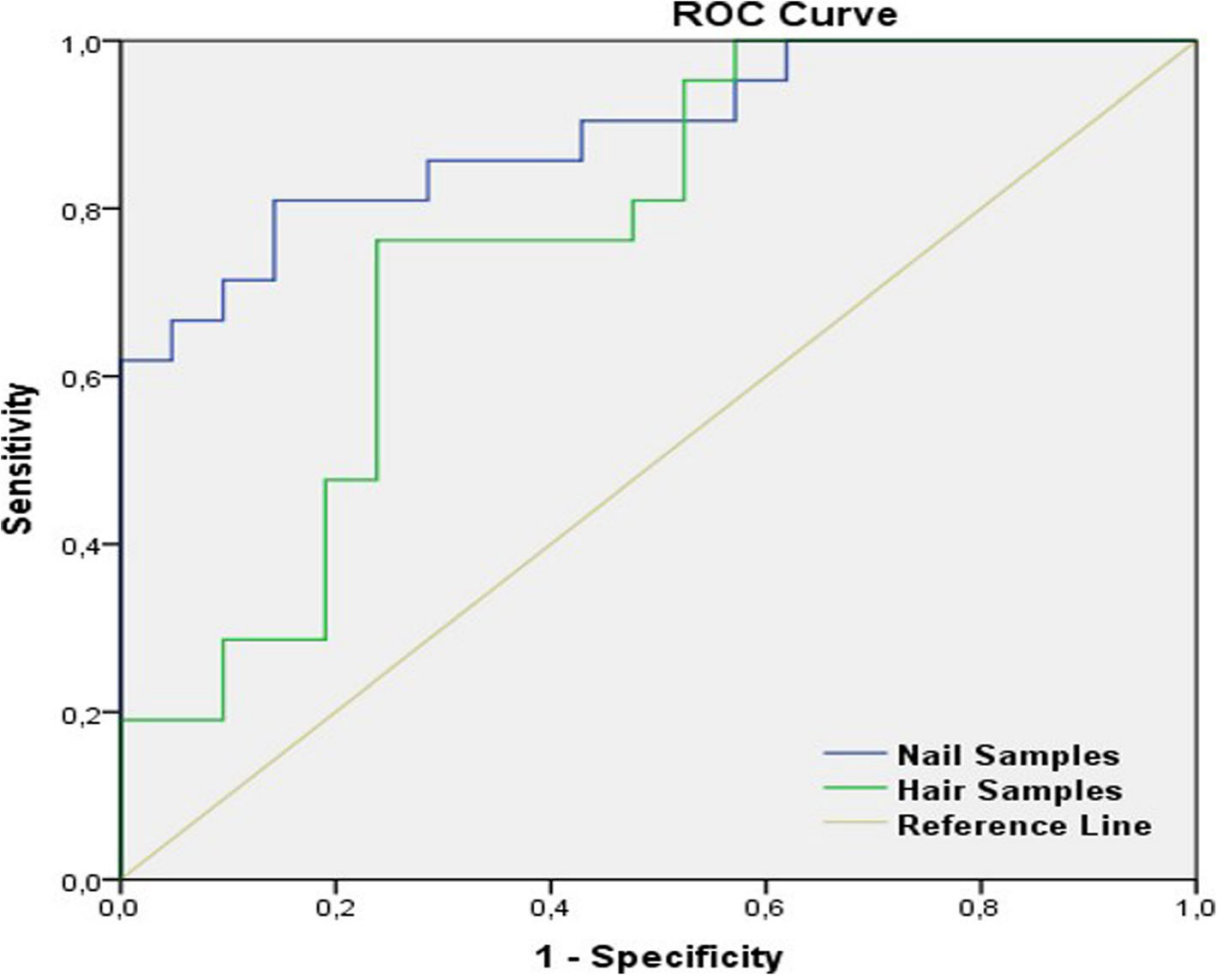
Receiver operator characteristic (ROC) curve analyses of big toenail and hair samples

The results of ROC analysis are given in Table 4 and Fig. 3. The relative positions of the plots indicate the relative accuracies of the tests. A plot lying above and to the left of another plot indicates greater observed accuracy. In Fig. 3, the fluoride assay for big toenails exhibits greater observed accuracy than does the fluoride assay for hair.

**Table 4:** Comparison of the area under the ROC curve (AUC) for fluoride concentration

Biomarker	AUC	Std. Error	P	95% Confidence Interval	
				Lower Bound	Upper Bound
Nail samples	0.889	0.050	< 0.001	0.791	0.987
Hair samples	0.762	0.075	=0.004	0.615	0.909

*ROC* Receiver Operating Characteristics, *AUC* Area Under Curve

The results of ROC analysis showed that the area under the curve (AUC) of big toenail samples was 0.889 (p < 0.001). The optimal sensitivity and specificity were 0.810 and 0.857, respectively (Table 4, Fig. 3). The AUC for hair samples was 0.762 (p = 0.004). The optimal sensitivity and specificity were 0.762 and 0.762 respectively (Table 4, Fig. 3).

## Discussion

The relationship between fluoride concentrations in big toenail (hallux) clippings, hair and the level of fluoride in the water of an endemic (IC) and non-endemic (SC) fluorosis region was assessed in this study.

The results of the present study, namely a higher and significant fluoride concentration in nail clippings and hair collected from IC participants when compared to SC participants, are similar to the findings of other studies [11–14]. These findings may be attributed to the fact that the regular level of fluoride in IC (≥2 ppm) drinking water was higher than that in SC (≤0.05 ppm) [18, 19]. Therefore, systemic fluoride circulation in the residents of IC is considerably higher, which in turn raised the fluoride uptake by nails and hair.

Absorbed fluoride deposits in the growing toenail by either continuous incorporation or secondary concentration [12]. One might argue that the detected level of fluoride in the nail clippings and hair collected from IC had a wide standard deviation indicating a significant individual discrepancy. This trend might be related to factors such as variation in the amount of consumed water [11–14], diet [14, 22], tea consumption [23] and external contamination among participants [24] as well as interpersonal variation in the amount of absorbed, circulated, metabolized and deposited fluoride [14].

The area under the ROC curve showed that nail and hair samples can serve as a biomarker to detect biological fluoride exposure according to the data of this pilot study. AUC is an effective way to summarize the overall diagnostic accuracy of a test [25]. In general, an AUC value of 0.7 to 0.8 is considered acceptable; whereas, an AUC value of 0.8 to 0.9 is considered excellent [26]. Thus, nail and hair samples have a reasonable discriminating ability to diagnose fluoride exposure. Nevertheless, hair is less sensitive and specific as a biomarker when AUC values of big toenail and hair samples are compared.

It is worth noting that the fluoride concentration for hair was lower than that for toenails. This outcome might be due to the fact that hair is characterized by a cyclic growth rate with different stages [27], whereas nails grow continuously and do not have a growth cycle analogous to that of hair [28]. In contrast, the fluoride concentration in toenails was higher than hair fluoride concentration and almost equal to the associated water. The growth rate of toenails [29] is substantially lower when compared to the growth rate of hair [30]. This lower growth rate of toenails might allow a more significant accumulation of fluoride. Furthermore, incorporation of fluoride through the nail bed, not only through the matrix (growth end), might contribute to the total fluoride concentration in toenails [3, 31].

## Conclusion

The null hypothesis was rejected, i.e. nail and hair samples can serve as biomarkers to detect biological fluoride exposure according to the data of this pilot study. Nail and hair samples have a reasonable discriminating ability to diagnose fluoride exposure from the water supply from an endemic and non-endemic fluorosis region. Nevertheless, hair is less sensitive and specific as a biomarker when AUC values of big toenail and hair samples were compared. This area merits further research with a larger sample size.

## Abbreviations

AUC: Area Under the Curve
cm: Centimeter
cm/month: Centimeter per month
HMDS: Hexamethyldisiloxane
IC: Isparta City
mg: Milligram
mg/kg: Milligram per kilogram
mm/day: Millimeter per day
Ppm: Parts per million
ROC: Receiver Operating Characteristics
SC: Samsun City
TFI: Thylstrup and Fejerskov Fluorosis Index
